# Effects of Polyphenols on the Structure, Interfacial Properties, and Emulsion Stability of Pea Protein: Different Polyphenol Structures and Concentrations

**DOI:** 10.3390/molecules30081674

**Published:** 2025-04-08

**Authors:** Shiyao Tang, Xiyuan Yang, Chang Wang, Changyuan Wang

**Affiliations:** College of Food, Heilongjiang Bayi Agricultural University, Xinfeng Road 5, Daqing 163319, China; 13212299708@163.com (S.T.); 13555517359@163.com (X.Y.)

**Keywords:** pea protein isolate, polyphenols, interfacial properties, emulsion stability

## Abstract

While protein-stabilized emulsions have demonstrated potential for various applications in food, their poor lipid oxidation remains a major challenge. The relationship between the architecture of polyphenolic compounds and their capacity to suppress lipid oxidation has not received extensive scrutiny. In this research, pea protein isolate (PPI)–polyphenol complexes were synthesized to examine their capability of maintaining emulsion stability and suppressing lipid oxidation. The collective evidence from fluorescence spectroscopy and molecular dynamics simulations pointed towards non-covalent and self-initiated interactions between the polyphenols and PPI. The presence of additional hydroxyl groups on the polyphenols could significantly boost the extent of these interactions. Specific clusters in PPI and polyphenols which might have formed hydrogen bonds and hydrophobic interactions. Polyphenols also reduced the interfacial tension and increased the surface hydrophobicity of the complex, thus driving more proteins to adsorb at the oil–water interface. The PPI-rosmarinic acid (RA)-stabilized emulsion had a smaller droplet size and higher electrostatic repulsion, enabling it to resist droplet aggregation. This emulsion stood out as having the most robust stability amongst all PPI-polyphenol emulsions and proved highly efficient in preventing lipid oxidation. This study bolsters the viability of employing polyphenol and pea protein-stabilized emulsions in developing new food products.

## 1. Introduction

The stability of emulsions is critical for their performance and shelf life. This stability is often achieved through the addition of emulsifiers [1]. Plant proteins have surged in popularity as viable alternatives to traditional animal proteins [2]. Among these, pea protein isolate (PPI), as a high-quality plant protein with abundant sources and low production costs, has a good amino acid profile and can meet the needs of human health. In addition, compared to mainstream soy protein, pea protein not only has no allergenicity but also maintains a better structural integrity at higher temperatures, making it more suitable for multi-scenario food-processing applications [3,4,5]. However, the compact structure of native PPI results in poor solubility and weak interfacial properties. Native PPI thus struggles to maintain the long-term stability of the emulsion system, which restricts its utility as an emulsifier. Therefore, it is necessary to modify PPI to improve its emulsification performance and expand its application in the field of emulsion.

Polyphenol is often used as a natural antioxidant in foods. Due to the presence of aromatic rings and abundant hydroxyl groups, polyphenols exhibit a high affinity for proteins [6]. Compared to enzymatic and chemical methods, protein–polyphenol non-covalent interactions provide proteins with new or enhanced functional properties through simple physical mixing [7]. Therefore, the potential for coupling polyphenols with proteins via non-covalent interactions to enhance the functional properties of proteins has become a research hotspot in recent years. Polyphenols can induce alterations to the three-dimensional structure of proteins, which inhibits the aggregation and flocculation of emulsion droplets and effectively improves the emulsification performance [8]. Gong et al. [9] demonstrated that young apple polyphenols promoted soy protein’s effective interfacial stacking, improving emulsions’ physical stability. Wang et al. [10] suggested that tannic acid and soy protein could form a non-covalent complex relieving the precipitation of the water phase in an emulsion and improve the emulsion stability. In addition, polyphenols can migrate to the oil–water interface, where lipid–protein co-oxidation occurs due to their high binding affinity with proteins, enabling polyphenols to exert their antioxidant properties effectively [11]. However, most studies have focused on the effect of the interaction between a protein and polyphenol on the emulsion performance, as studied by varying the polyphenol’s concentration, molecular weight, and environmental conditions (such as pH and T) [6,12]. Yet, the hydroxyl groups and carbon chain length in polyphenols are key functional groups that drive non-covalent interactions with proteins, and the structure directly affects the affinity between proteins and polyphenols [8]. Currently, there is a lack of literature investigating the interaction mechanisms between polyphenols with different structures and pea proteins, and their impact on the emulsifying properties of pea protein. Caffeic acid (CF) and rosmarinic acid (RA) both play important roles in improving human health through dietary and pharmaceutical supplements. In addition, both CF and RA are considered to have good antioxidant activity and can effectively inhibit lipid oxidation.

In this study, we investigated the binding ability of RA and CF with PPI. The effect of the polyphenolic structure on the binding ability and conformational alterations between PPI and polyphenols at multiple spatiotemporal scales was studied via spectroscopy, molecular docking, and molecular dynamics simulations. The changes in the interfacial tension and interfacial protein content (AP%) of the complexes at the oil–water interface were observed. The emulsion characteristics of PPI-polyphenol stabilized emulsions were comprehensively studied through an array of techniques, including emulsion droplet size measurement, the Zeta potential, and confocal laser scanning microscopy (CLSM), as well as rheology. Finally, the physical stability and oxidative stability of as-prepared emulsions were systematically studied. The findings offer crucial insights into the interplay between protein and polyphenols and will guide the development of emulsions with higher oxidative stability.

## 2. Results

### 2.1. Fluorescence Quenching

**Figure 1:**
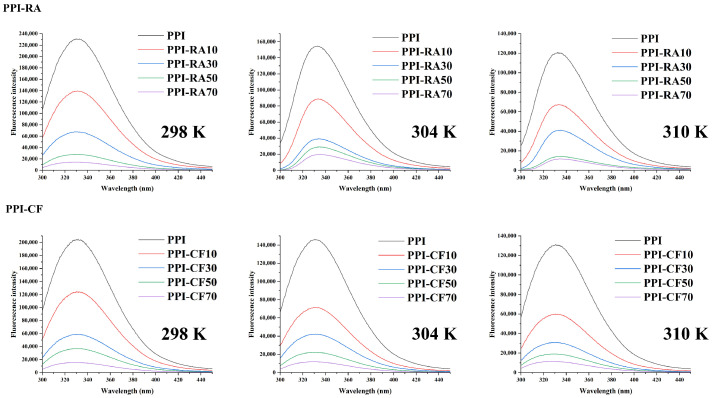
Fluorescence quenching of PPI-RA and PPI-CF.

The fluorescence quenching effect can be used to investigate the conformational changes in PPI after adding polyphenols at different concentrations [13]. From Figure 1, we can find that upon excitation at a wavelength of 280 nm, the maximum emission wavelength of PPI was 320 nm. PPI that did not react with polyphenols had the strongest fluorescence emission intensity of TYR and TRP. After adding RA or CF, the fluorescence intensity of PPI decreased, and with the increasing concentration of polyphenols, the emission fluorescence intensity showed a gradually decreasing trend. These findings suggested that after adding polyphenols, the protein structure progressively unfolded and displayed more binding area near TYR and TRP residues to match polyphenols [14]. To delve deeper into the quenching mechanism and differences in the interaction between polyphenols and PPI, Stern–Volmer plots were used for our research. As shown in Table 1, for the same polyphenol, with the increasing temperature, a decreasing trend of Ksv was shown. This indicated that the quenching types of RA and CF to PPI were both static, and these polyphenols could bind with PPI to form a complex and quench protein fluorescence. Compared with PPI-CF, PPI-RA exhibited a larger Ksv. RA was more likely to induce the structural unfolding of PPI, allowing more energy to be transferred from the excited state of PPI fluorophores to RA molecules. Ultimately, more stable complexes were formed, and RA showed a stronger ability to quench PPI fluorescence.

The binding affinity between polyphenols and PPI was assessed utilizing the double logarithmic form of the Stern–Volmer equation. As shown in Table 1, Ka and n gradually increased with the increasing temperature, and PPI-RA had higher values compared with PPI-CF. RA had more active phenolic hydroxyl functional groups, which provided more possibilities for forming hydrogen bonds between RA and PPI. In addition, RA had a longer carbon chain, exhibiting a more flexible and easily twisted topological structure. In the process of binding with PPI, RA was more likely to enter PPI by changing its own structure and forming small molecule structures that were more compatible with protein hydrophobic pockets, thereby increasing the binding strength [15]. Therefore, PPI-RA had higher n and Ka. To further ensure the main binding force types between polyphenols and PPI, thermodynamic parameters (ΔG, ΔH, and ΔS) were derived using the Van’t Hoff equation. As shown in Table 1, ΔG < 0 indicated that there was spontaneous binding at RA/CF and PPI. For ΔH and ΔS, when ΔH > 0 and ΔS > 0, hydrophobic interaction was the main binding force [16]. As the ΔH and ΔS of PPI-RA/CF exhibited positive values, we inferred that hydrophobic interactions could occur between aromatic/aliphatic side chains in proteins and phenolic aromatic rings, and these interactions were considered to be the main forces for producing PPI-polyphenol complexes.

### 2.2. Molecular Docking

**Figure 2:**
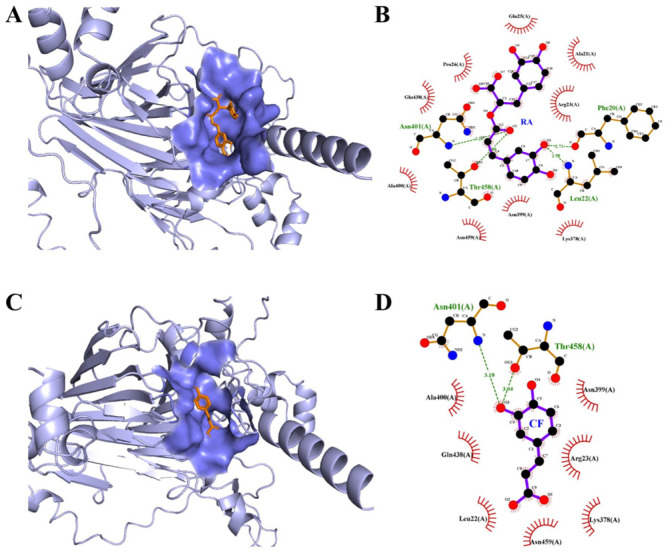
Best 3D docking positions of PPI-RA (**A**) and PPI-CF (**C**); 2D view diagrams of PPI-RA (**B**) and PPI-CF (**D**).

Molecular docking techniques were employed to simulate the non-covalent binding of polyphenols and PPI and calculate their binding affinities [17]. In the results, the binding affinity between PPI and polyphenols was predicted to be RA (−7.3 kcal/mol) > CF (−5.6 kcal/mol), which was consistent with the results of the binding constant. Figure 2 shows the best docking positions of polyphenols and PPI. Figure 2A,B show that both polyphenol molecules could enter the hydrophobic pocket of PPI by twisted their structure, forming PPI-polyphenol complexes with complementary structures. As shown in Figure 2B, two hydrogen bonds were formed through oxygen atoms in ester bonds (O_2_) of RA and ASN 401/THR 458 of PPI, and other two hydrogen bonds were formed through O_3_ on phenolic hydroxyl groups of RA and PHE20/LEU22 of PPI. Many amino acid residues of PPI, such as ARG23, ALA21, GLN25, PRO26, GLN438, ALA400, ASN459, ASN399, and LYS378, could form hydrophobic pockets to interact with RA. However, for CF, only two hydrogen bonds could be formed through O_3_ on phenolic hydroxyl groups of CF and ASN401/THR458 of PPI. In addition, less amino acid residues of PPI could interact with CF through hydrophobic interaction. The strength of hydrogen bonds had a direct positive relationship with the quantity of hydroxyl groups [8]. These results indicated that RA with more hydroxyl groups had higher molecular activity and affinity, so it could bind strongly with protein through more hydrogen bonds. In addition, longer carbon chains gave RA a more flexible conformation, making it easier to adjust its structure to form a complementary topological structure. Therefore, compared with CF, RA showed a higher binding ability with PPI. However, molecular docking technology could not provide more detailed information on the binding process between proteins and polyphenol. Subsequently, MD simulations were conducted to investigate the changes in PPI and RA/CF over a time period of 100 ns.

### 2.3. MD Simulation

**Figure 3:**
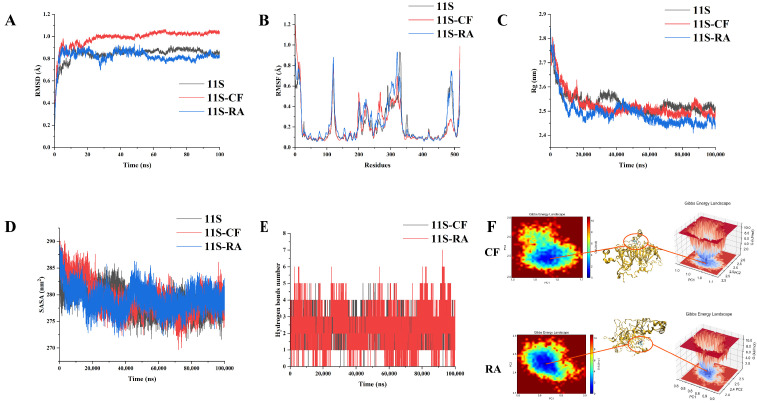
Time evolution of 11S-polyphenol complex for their RMSD (**A**), RMSF (**B**), Rg (**C**), SASA (**D**), hydrogen bond number (**E**), and Gibbs energy landscape (**F**).

Further analysis of protein and two polyphenols’ binding was conducted through molecular dynamics simulation. The RMSD was the overall amplitude of protein movement [18]. The larger the RMSD, the larger the spatial range of protein skeleton movement. Broadly speaking, a simulation system could be deemed to have reached an equilibrium condition when the fluctuations in the mean RMSD value remained below 1.0 nm. Figure 3A illustrates that the average RMSD of the protein backbone without (11S) and with polyphenols (11S-CF/RA) showed an upward trend during the 0-60 ns period, followed by equilibrium. This suggested that all complexes achieved a relatively stable conformation state after 100 ns. For 11S-CF, the RMSD was higher than 11S and even exceeded 1.0 nm. However, 11S-RA showed a lower RMSD of the protein backbone, and it fluctuated around 0.78 Å. The shorter carbon chain and specific hydroxyl positions of CF limited its rotational freedom, reducing its adaptability and flexibility in interacting with proteins. Therefore, during the combination of 11S with CF, the skeleton structure of the protein experienced significant fluctuations and ultimately an easily broken complex was formed. The long carbon chain structure of RA had higher flexibility, allowing it to adjust its shape through chain twisting and rotation, thus better adapting to complex structures on the surface or inside of proteins. This characteristic allowed RA to penetrate deep into the hydrophobic core of proteins and form stable complexes with the protein’s interior. The degree of freedom of protein movement was decreased [19]. The relatively low RMSD values of 11S-RA revealed the stability and good binding of proteins throughout the simulation period.

The RMSF is used to evaluate the migration rate of local proteins after binding to polyphenols [20]. The RMSF of most residues in the protein experienced significant fluctuations after binding to polyphenols (Figure 3B). This may be due to the protein structure stretching and secondary structure changing of 11S during its binding with polyphenols, resulting in differences in the positions of some amino acid residues. The increased levels of amino acids in RMSF stabilized the amino acid flexibility of hydrophobic pockets. On the contrary, the decrease in RMSF was due to the instability of the complexes caused by the binding of the complexes on the hydrophobic surface rather than on the hydrophobic pocket. Therefore, it could be found that the structure of the protein experienced complex processes of changes.

The radius of gyration (Rg) serves as a metric to assess the global compactness of the complexes’ structures formed when 11S interacts with polyphenols [21]. From Figure 3C, it can be seen that the Rg of 11S was reduced at the beginning of simulation and then fluctuated within a small range until the end of the simulation. After binding with polyphenols, the Rg decreased, and the average Rg value of the 11S-RA complexes had a slightly lower value. This might be due to the insertion of CF/RA into the interior of 11S, causing the protein to expand outward. This indicated that during the process of binding with polyphenols, the protein density decreased and the structure gradually transitioned from tightly folded to a loose state. After inserting CF/RA into the interior of 11S, a denser surface composite was formed again. RA could bind with the protein to form tighter complexes. Figure 3D presents the solvent-accessible surface area (SASA) values for 11S. Throughout the simulation process, the SASA values of 11S and 11S-CF/RA complexes were almost identical. This indicated that the two selected polyphenols did not have a strong impact on the SASA fluctuation of 11S. The primary reason was the interaction occurring between 11S and polyphenols, which mainly caused internal regional structural movement rather than significant changes in its overall structure.

To further ensure the interaction between 11S and polyphenols, the time dependence of the number of hydrogen bonds (Figure 3E) between 11S and CF/RA was determined. During the entire 100 ns dynamic simulation process, most hydrogen bonds between 11S and RA remained essentially unchanged in the range of 5–6. This indicated that hydrogen bonds had strong and stable binding throughout the simulation process, with the most hydrogen bonds between 11S and RA. CF had a catechol structure with three hydroxyl groups, which limited its ability to form hydrogen bonds. As a derivative of a CF dimer, RA had four hydroxyl groups and one carboxylic acid group, and these hydroxyl groups were distributed on longer carbon chains. Therefore, RA was more likely to form a hydrogen bond network with multiple structural domains of 11S by changing its own carbon chain, enhancing the strength of the interaction between the two. This was consistent with the results of the binding constant (Ka) and the number of binding sites (n) in Section 2.1. Due to the formation of more hydrogen bonds between RA and the protein, their n and Ka values were higher, indicating a tighter and more stable binding between the two. This stronger interaction was more conducive to improving the stability of PPI. In addition, the Gibbs energy landscape of complexes (Figure 3F) also showed that the free energy of the 11S-RA complexes was lower, and this complex represented the most stable state.

### 2.4. Surface Hydrophobicity (H_0_)

**Figure 4:**
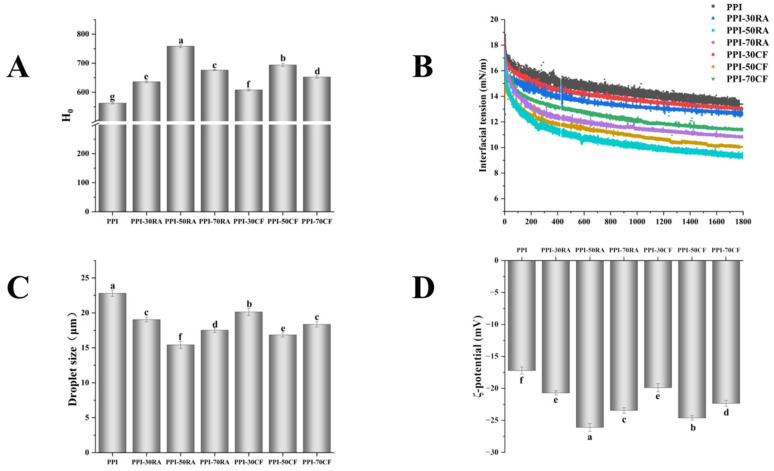
The surface hydrophobicity (**A**) and interfacial tension (**B**) of PPI-RA and PPI-CF, and the droplet size (**C**) and ζ-potential (**D**) of emulsions stabilized by PPI-RA and PPI-CF. Note: Values with a different letter(s) indicate a significant difference at *p* ≤ 0.05.

H0 represents the exposure of hydrophobic groups on the protein surface and has a strong correlation with the interfacial behavior of proteins. Figure 4A depicts the H_0_ trend of PPI-RA and PPI-CF complexes. The H_0_ of PPI first increased and then decreased as the polyphenol concentration increased. The H_0_ value peaked when the polyphenol concentration reached 50 mM, suggesting that at this level of condition, polyphenols caused the PPI structure to be more completely unfolded. These phenomena were attributed to the formation of hydrogen bonds between carbonyl groups of PPI and the hydroxyl groups of RA and CF. This interaction decreased the exposure of hydrophilic groups to the external environment, leading to an increase in hydrophobicity of PPI. However, when the concentration of polyphenols reached 70 mM, excessive polyphenol molecules increased the number of polar groups such as hydroxyl and carboxyl groups, causing a change in the polarity of the protein surrounding environment, which increased its hydrophilicity. The PPI-RA complexes had higher surface hydrophobicity than PPI-CF complexes, indicating that the surface of PPI-RA molecules has more hydrophobic groups and the degree of molecular structure stretching is greater. The reason might be that compared to CF, RA contained more hydroxyl groups and could form more hydrogen bonds with PPI, making the protein surface more hydrophobic. The change in hydrophobicity of the PPI surface might be one of the main reasons for the alteration of its emulsifying properties.

### 2.5. Emulsifying Properties

Emulsifying properties can be used to macroscopically evaluate the ability of PPI-polyphenol complexes in stabilizing the oil–water interface [22]. The effects of different concentrations and types of polyphenols on the EAI and ESI of PPI are shown in Table 2. During the increase in polyphenol concentration, the values of both the EAI and ESI first increased and then decreased, with the maximum emulsifying property being observed at a polyphenol concentration of 50 mM. In addition, the PPI-RA complex had a higher emulsifying property than PPI-CF at any polyphenol concentration. Appropriate concentrations of polyphenols could interact with amino acid residues in proteins through the large number of hydroxyl groups, further inducing the extension of protein spatial structure and the appearance of loose conformation in proteins [23]. Exposure of the internal hydrophobic groups made protein easier to diffuse to the oil–water interface, thus reducing the energy barrier and increasing EAI and ESI. However, the high concentration of polyphenols could become a bridging agent for proteins or protein–polyphenol complexes, promoting the gradual formation of dimers or polymers with a tight structure [24]. This insoluble aggregate hindered the formation of the oil–water interface, reducing the ability of proteins to unfold at the interface and leading to a decrease in EAI and ESI. The phenolic hydroxyl groups in polyphenols were the main binding sites and key factors for interactions with proteins. Compared with CF, RA had more phenolic hydroxyl groups, so there were more binding sites in the protein that could interact with RA. RA was more likely to induce protein conformational stretching. This led to a progressive transformation of the protein structure from an ordered to a more disordered state, enhancing the amphiphilic properties of protein. In addition, based on the results of MD simulation, the denser hydrogen bonding network between RA and protein resulted in a tighter arrangement of the RA-PPI complex at the interface, forming a protective film to prevent droplet aggregation and enhance ESI. Therefore, the EAI and ESI of PPI-RA were higher.

### 2.6. Interfacial Tension

Dynamic interfacial tension, a key parameter tied to the assembly characteristics of proteins at the oil–water interface, is vital for both the formation and stabilization of emulsion systems [25]. From Figure 4B, it can be seen that the interfacial tension decreased over time, suggesting that the protein adsorption at the oil–water interface was a dynamically evolving process. Compared with the interfacial tension of PPI, those of all complexes decreased. In addition, as the concentration of polyphenols increased, the interfacial tension showed a trend of first decreasing and then increasing, and PPI-RA showed lower interfacial tension. When 50 mM RA was added, the interfacial tension decreased from an initial value of 18 mN/m to 9.2 mN/m within 1800 s, which showed the higher rate of decrease in interfacial tension. PPI, being a globular protein, demonstrated a weak capability in stabilizing interfaces [26]. RA and CF facilitated the unfolding of PPI, which exposed more hydrophobic groups and augmented the interaction between the protein and the oil phase. This meant PPI could quickly adsorb to interface and decreased the interfacial tension. Higher concentrations of polyphenols could promote this effect. When the concentration of polyphenols reached 70 mM, excessive modification of PPI by polyphenols led to a large number of hydrophobic amino acids participating in protein binding, resulting in large and complex aggregates. Therefore, the interfacial tension of complexes decreased. For the same concentration of polyphenol, the strong interaction between RA and PPI promoted a looser and more dissociated protein structure, endowing the PPI-RA complexes with better flexibility and fluidity. This promoted faster diffusion, permeation, and rearrangement of the complex at the interface, thus lowering the interfacial tension. Combined with the results of MD simulation (Figure 3), we surmise that RA with more –OH groups could achieve tight binding with proteins through multiple hydrogen bonds. The stable RA-PPI complex reduced the energy consumption of conformational rearrangement during interfacial adsorption, allowing proteins to anchor faster at the oil–water interface and reducing interfacial tension.

### 2.7. Interfacial Protein Content (AP%) of Emulsion

The interfacial protein content (AP%) of the emulsion determined the key factors contributing to emulsion properties. As shown in Table 2, compared with emulsion stabilized by PPI, the AP% increased after adding the polyphenols. With the increasing polyphenol concentration, the AP% of the emulsion stabilized by the PPI-RA complex and PPI-CF complex initially rose and then declined, reaching their maximum values at an RA concentration of 50 μM. Polyphenol molecules with higher concentrations could induce the protein structure to open, and enhance the structural flexibility. More hydrophilic and lipophilic groups were exposed, promoting more proteins to adsorb to the oil–water interface. Therefore, the AP% increased [27]. However, when the concentration of polyphenols was too high, many polyphenols were bound to PPI, and proteins could be bridged by excess polyphenols, forming large particle aggregates of protein–polyphenol–protein [28]. Highly aggregated molecules encapsulated more hydrophobic amino acid residues internally, making it difficult to adsorb at the oil–water interface. Compared with PPI-CF, PPI-RA showed a higher AP%. RA with more hydroxyl groups and a flexible topological structure could bind to PPI through stronger interaction forces, which enhanced the electrostatic interactions, ensuring the stability of individual proteins at the oil–water interface [29]. The RA molecule contains five hydroxyl groups uniformly distributed on the biphenyl propane skeleton, which promotes the formation of more hydrogen bonds and interaction points between RA and the protein surface, helping to improve the overall structure and hydrophilic hydrophobic balance of the protein. The optimized PPI-RA complex can move more efficiently in the emulsion system and locate at the oil–water interface. Therefore, the hydroxyl distribution of RA not only enhances its binding ability to protein but also promotes the effective migration of the complex in an emulsion. In contrast, while caffeic acid (CF) also contains three hydroxyl groups, these hydroxyl groups are mainly concentrated on a benzene ring, which limits its interaction with the protein surface and prevents it from forming a wide range of connection points like RA. Therefore, the AP% of emulsion prepared by PPI-RA was higher than PPI-CF.

### 2.8. Property of Emulsion Prepared by PPI-Polyphenol

#### 2.8.1. Droplet Size and Zeta Potential

Generally, emulsions with smaller droplets tend to exhibit greater stability. Figure 4C displays the droplet size results of different emulsions. With the increase in polyphenol concentration, the droplet size first decreased and then increased. In addition, the emulsion stabilized by PPI-RA showed a smaller droplet size, suggesting that an increase in the number of hydroxyl groups from polyphenols was more conducive to forming a smaller droplet size. The composite of appropriate concentrations of polyphenols could induce more PPI to involve the formation of a three-dimensional interfacial network, effectively preventing oil droplets from coalescing. However, excessive hydroxyl groups might cause the formation of larger protein aggregates, making it difficult to unfold at the O/W interface of the emulsion, which was unfavorable for maintaining the stability of the emulsion [30]. When the polyphenol concentration was consistent, the emulsion stabilized by PPI-RA exhibited a smaller droplet size.

The Zeta potential is an important index to characterize the stability of an emulsion. With a smaller absolute value of the Zeta potential, the molecular attraction in the system exceeds the repulsive force, and the molecules tend to flocculate or aggregate. As shown in Figure 4D, all sample Zeta potentials were negative, a result attributed to the negatively charged surfaces of PPI molecules when the pH surpassed their isoelectric points. With the increase in polyphenol concentration, the absolute value of the Zeta potential of the emulsion showed the tendency of increasing firstly and then decreasing, and emulsions stabilized by PPI-50RA and PPI-50CF reached the maximum. This phenomenon is likely due to the increased electronegativity and subsequent electrostatic repulsion caused by polyphenols binding to the adsorbed proteins, which significantly enhances the charge on the droplet surface. It was intriguing to note that when the polyphenol concentration was raised further, there was a subsequent reduction observed in the magnitude of the electrical charge in the emulsion surface. The reason for this phenomenon might lie in the capacity of high polyphenol concentrations to modify the protein structure adsorbed on the interface through either non-covalent or covalent interactions, thus changing the locations of the charged groups. The Zeta potentials of PPI-50RA and PPI-50CF were −26.06 and −24.59 mV, respectively, suggesting that polyphenols possessing a greater number of hydroxyl groups are more effective at modifying the structure of PPI, facilitating the exposure of its internal charged groups. Colloidal particles with stronger repulsions were less prone to aggregate owing to the interactions between particles. This led to an elevated number of particles adsorbing at the oil–water interface, thereby promoting the formation of a more stable emulsion.

#### 2.8.2. Confocal Laser Scanning Microscope (CLSM)

**Figure 5:**
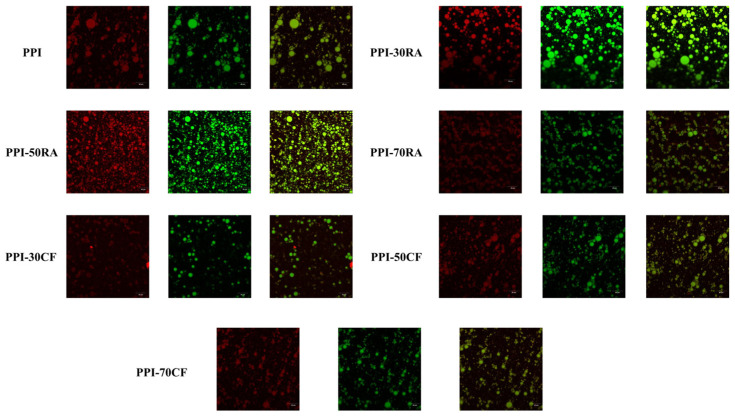
CLSM of emulsions stabilized by PPI-RA and PPI-CF. The aqueous phase was labeled with Nile red (in green), and the oil phase was labeled with Nile blue (in red). The scale size is 40 μm.

The particle distribution, continuous phase network structure, and aggregation state of the composite system of a protein polyphenol emulsion were observed by CLSM (Figure 5). Among the colors, red represents the composition of the oil phase, and green represents the protein. Emulsions stabilized by PPI showed a nearly spherical droplet structure. With the increase in polyphenol concentration, the size of emulsion droplets gradually decreased and the distribution became more uniform. When the concentration of polyphenols reached 70 mM, the oil droplets reaggregated and exhibited large-scale flocculation. In addition, the emulsion stabilized by PPI-RA showed more uniform droplets with smaller particle sizes. Due to the limitation of a spherical structure, natural PPI particles were not excellent stabilizers for emulsion [31]. However, PPI and polyphenols could be connected through forces such as hydrogen bonding and hydrophobic interactions, inducing a more open protein structure. The steric hindrance effect between droplets increased, to form a stable and uniform emulsion. Consequently, incorporating polyphenols could enhanced the wettability of PPI and diminish interfacial tension, which fostered swift adsorption at the oil–water interface [8]. However, when the concentration of polyphenols reached 70 mM, excessive polyphenol molecules accumulated on the surface of the protein. The PPI-polyphenol complexes which adsorbed on the interface squeezed each other, causing fluctuations in the interface layer and an increase in surface tension. Therefore, a large number of oil droplets was accumulated. The composite formed by PPI and RA prepared a more uniform and stable emulsion system. From the previous results, it could be concluded that RA could induce a greater degree of unfolding of PPI, and a more flexible structure was more conducive to achieving diffusion, adsorption, and rearrangement at the interface. In addition, RA had more hydroxyl groups and could form more hydrogen bonds with PPI at the interface, promoting the formation of a more stable and compact interfacial membrane. Therefore, the PPI-RA-stabilized emulsion is more uniform and stable.

#### 2.8.3. Rheological Behaviors

**Figure 6:**
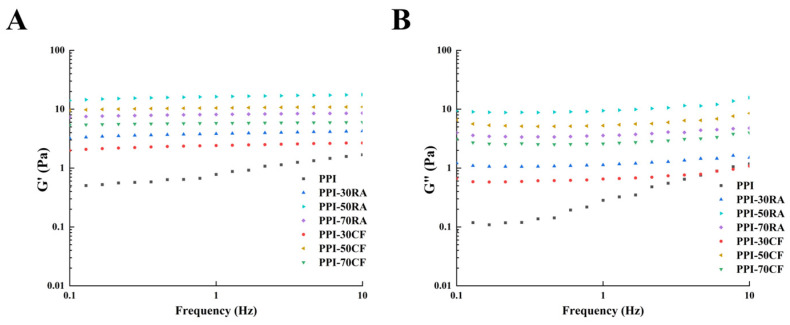
Frequency sweep of emulsions stabilized by PPI-RA and PPI-CF. (**A**) G′ (storage modulus) and (**B**) G″ (loss modulus).

In order to examine how PPI in combination with different types of polyphenols impacts the rheological properties and opposition to deformation of emulsions, measurements of G′ (storage modulus) and G″ (loss modulus) were conducted, with the results depicted in Figure 6. In the test range, G′ was always higher than G″ for all emulsion samples, which indicated that the emulsion samples exhibited elastic gel-like behavior. The values of both G′ and G″ for PPI-polyphenol complexes exceeded those of PPI alone, indicating that the incorporation of polyphenols led to an enhancement in the rheological characteristics of emulsions. Additionally, it was observed that the G′ and G″ values for emulsions containing PPI-polyphenol complexes exhibited a trend of initially rising followed by a decline as the concentration of polyphenols was increased. In particular, PPI-polyphenol had the highest G′ and G″ values at the concentration of 50 mM. This observation was credited to the formation of a gel-like network structure composed of smaller and more compact droplets, which endowed the system with high viscoelasticity and firmness to effectively resist some stress and strain [32]. However, upon reaching a polyphenol concentration of 70 mM, both G′ and G″ values showed a decline. This was because the excessive polyphenol could form large particle aggregates with PPI, thereby making it difficult to adsorb at the oil–water interface, which decreased surface particles of droplets and damaged the network structure of the emulsion. Rheological results indicated that the PPI-polyphenol complexes with suitable concentrations and more hydroxyl groups could stabilize the emulsions and generate excellent viscoelasticity.

#### 2.8.4. Stability of Emulsion

##### Emulsion Stability

**Figure 7:**
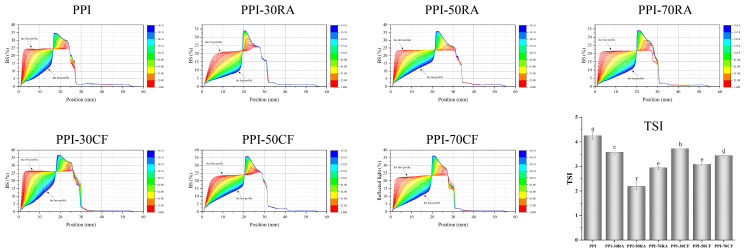
Backscattering profiles and TSI values of emulsions stabilized by PPI-RA and PPI-CF. Note: Values with a different letter(s) indicate a significant difference at *p* ≤ 0.05.

Emulsion stability is an important factor that affects the shelf life of emulsified foods. The stability of the emulsions was evaluated using a Turbiscan. Figure 7 shows the BS profiles and TSI results of the emulsions. The BS profiles reflected the macroscopic characteristics of the emulsions, making it possible to study droplet movement and predict the stability of the emulsions [33]. At the initial stage, the BS spectrum of the PPI-stabilized emulsion showed almost flat curves at the bottom, middle, and top. With the extension of the storage time, the BS spectral curve at the bottom and middle stages rose rapidly, and a convex peak appeared at the top (17 mm), which indicated that the emulsion stabilized by only PPI was prone to flocculation and stratification, and the emulsion had poor stability. When polyphenols were added, the convex peak of the BS spectrum shifted to the right as a whole, and the trend of the right shift of the convex peak gradually increased. When the concentration reached 70 mM, the convex peak began to shift to the left. This indicated that the addition of polyphenols significantly improved the storage stability of emulsions. The non-covalent adsorption of polyphenols onto PPI could form a thick layer, resulting in a steric hindrance effect and electrostatic repulsion, thereby improving emulsion stability [34]. When the concentration of polyphenol reached 70 mM, the protein membrane collapsed and the stability of the emulsion deteriorated due to the aggregation of the PPI-polyphenol complex at the interface adsorption site and the decrease in its amphiphilicity. In addition, it can be found that compared with PPI-RA, the BS peak position of the emulsion prepared by PPI-CF was on the left, indicating that the layering of the emulsion prepared by the PPI-CF complex was more serious. This was consistent with the result for the emulsion particle size.

The analysis of the BS profile data was employed to determine the TSI, thus suggesting emulsion stability and phase separation. The lower the TSI value, the higher the stability of emulsion [35]. The TSI value of the emulsion after adding polyphenols was significantly reduced, and the emulsion prepared by the PPI-RA composite had a lower TSI.

##### Emulsion Oxidative Stability

**Figure 8:**
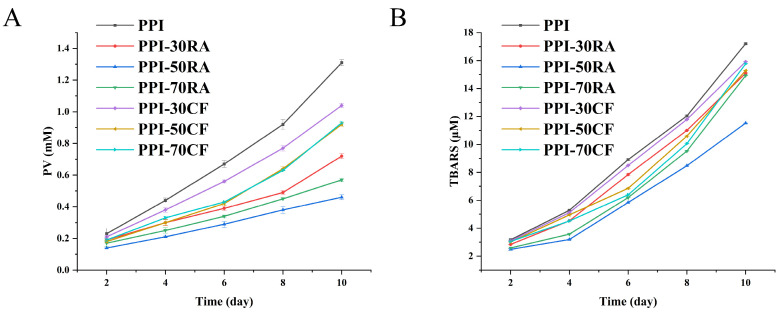
PV values (**A**) and TBARS values (**B**) of the emulsions stabilized by PPI-RA and PPI-CF during 10 days of storage.

The evolution of primary and secondary oxidation by-products was quantified in the emulsion over a 10-day storage period, providing an initial assessment of lipid oxidation within the emulsion (Figure 8). As the storage duration was extended, the manifestation of lipid oxidation intensified, resulting in a swift escalation in both PV and TBARS levels across all the emulsions. However, compared with PPI, the content of oxidation products in emulsions stabilized by PPI-polyphenols decreased in the same storage days. With the increase in polyphenol concentration, the content of oxidation products showed a trend of first increasing and then decreasing. Furthermore, the emulsions that were solely stabilized by PPI displayed the most rapid rate of lipid oxidation. Conversely, incorporating both polyphenols led to a deceleration in the oxidation process, with the PPI-50RA complex demonstrating the most potent inhibitory effect. This protective action could be ascribed to the intrinsic antioxidant properties of the polyphenols, which have the capacity to disrupt free radical chain reactions and diminish the concentration of highly reactive low-valence metal ions, thereby directly thwarting lipid oxidation [36]. In comparison to the PPI-CF complex, the incorporation of RA brought about a higher number of hydroxyl groups. RA could more effectively provide hydrogen atoms or electrons, thus neutralizing these reactive oxygen species (ROS) and preventing them from further attacking the oil molecule. In addition, RA’s catechol and carboxylic acid groups could synergistically chelate metal ions, while CF only binds to metal ions through catechol hydroxyl groups, with a limited inhibitory effect on metal-catalyzed oxidation. Combining the results of interfacial tension and AP% allows us to surmise that PPI-50RA could form a denser, continuous, and stable interfacial film. This dense interface film helped to block oxygen from reaching the interior of the oil phase, thereby reducing the possibility of lipid oxidation [37]. Additionally, the emulsion stabilized by PPI-50RA with a smaller particle size might also contribute to its higher oxidative stability. However, high concentrations of polyphenols might cause excessive cross-linking of proteins and weaken the interfacial layer, which induced the decrease in oxidative stability. Taken together, these results underscore that PPI-RA exhibited an enhanced oxidative stability when forming an emulsion. This increased oxidative stability holds promise for expanding the application scope of emulsions stabilized by PPI.

## 3. Materials and Methods

### 3.1. Materials

The pea protein isolate (PPI) with 90% protein on a dry matter basis was obtained from Guangzhou Yibaolai Biotechnology Co., Ltd. (Guangzhou, China). Rosmarinic acid (RA, ≥97%, CAS: 20283-92-5), caffeic acid (CF, ≥98%, CAS: 331-39-5), Nile Blue, and Nile Red were obtained from Yuanye Bio-tech Co., Ltd. (Shanghai, China). Sunflower oil was purchased in local supermarkets (Harbin, China) and produced by COFCO Fulinmen Food Marketing Co., Ltd. (Tianjin, China). All additional chemical agents used were of analytical grade.

### 3.2. Preparation of PPI-Polyphenol Complexes

The PPI and polyphenol solutions were prepared using a phosphate buffer solution (PBS, KH_2_PO_4_/K_2_HPO_4_, 0.01 M, pH 7.0) at 25 °C. The PPI-RA/CF complexes were fabricated by combining PPI with each specific polyphenol in a suitable volume ratio, with the aim of attaining a final PPI concentration of 10 mg/mL alongside polyphenol concentrations of 30 μM, 50 μM, and 70 μM, respectively. The different complexes were named PPI-30RA, PPI-50RA, PPI-70RA, PPI-30CF, PPI-50CF, and PPI-70CF.

### 3.3. Fluorescence Spectroscopy

Solutions were prepared with a PPI concentration of 1 mg/mL, and polyphenol concentrations were systematically adjusted to 0, 10, 30, 50, and 70 μM. The spectra were acquired using an F-7000 fluorescence spectrophotometer (Shimadzu, Tokyo, Japan). The recordings took place at 298 K, 304 K, and 310 K, covering the spectrum between 300 and 450 nm, and an excitation wavelength of 280 nm was selected.

To delve deeper into understanding the quenching dynamics between PPI and the RA/CF compounds, the Stern–Volmer equation was used.

$$\frac{F_{0}}{F}=1+K_{q}\tau_{0}\left[Q\right]=1+K_{SV}\left[Q\right]$$

Here, F denotes the fluorescence intensity of PPI-RA/CF, whereas F_0_ signifies the intensity of PPI. K_q_ represents the bimolecular quenching reaction rate constant, and τ0 is the lifetime of fluorophore when no quencher is present. [*Q*] is the concentration of the RA/CF, and K_SV_ is identified as the Stern–Volmer constant.

To ascertain the binding constant and the number of binding sites, one could utilize the subsequent equation:

$$log\left[\frac{F_{0}-F}{F}\right]=logK_{a}+nlog\left[Q\right]$$

Here, K_a_ and n are the binding constant and number of binding sites, respectively.

### 3.4. Thermodynamic Parameters

The interaction types can be determined based on thermodynamic analysis. If the change in ΔH is not significant over a certain temperature range, the value of ΔS can usually be calculated using the Van’t Hoff equations as follows:

$$lnK_{a}=-\frac{\Delta H}{RT}+\frac{\Delta S}{R}$$

Here, ΔH represents the alteration in enthalpy (kJ·mol^−1^), and ΔS signifies the modification in entropy (J·mol^−1^). R is 8.314 J/(mol·K) and T is the temperature in Kelvin. Furthermore, the variation in Gibbs free energy (ΔG), a crucial thermodynamic parameter, can be ascertained by applying the subsequent equation:

ΔG=ΔH−TΔS

### 3.5. Molecular Docking

The AutoDock Vina 1.1.2 software was utilized to perform the molecular docking simulations. Specifically, the 11S pea protein structure (PDB ID: 3KSC), which is the major protein component found in PPI, was chosen as the model for the study. The three-dimensional configurations of rosmarinic acid (RA, CID: 5281792) and caffeic acid (CF, CID: 689043) were obtained from the PubChem database.

### 3.6. Molecular Dynamics (MD) Simulations

Molecular dynamics (MD) simulations were carried out for both PPI alone and the PPI-polyphenol complex using GROMACS 2022. The topological parameters of proteins and ligands were generated by AMBER99SB-ILDN using an all-atom force field in GROMACS and AMBER14SB force field on the website of ACPYPE, respectively. Initially, the periodic boundary condition was applied and a cubic box was placed around all atoms with a distance of 10 Å. TIP3P water molecules with a density of 0.10 g/mL were used to solvate the system. Na^+^ or Cl^−^ was added to neutralize the charges in the system. The steepest descent method was used to minimize energy. After energy minimization, regular systematic simulation (NVT, 2 ns) followed by isothermal and isobaric simulation (NPT, 1 ns) was performed to ensure MD operation of the system at a constant temperature and pressure (298 K, 1 bar) for 100 ns.

### 3.7. Surface Hydrophobicity (H0)

In summary, a solution of 8-anilino-1-naphthalenesulfonic acid (ANS) at a concentration of 8 mmol/L was prepared. Samples were then diluted to attain concentrations within the range of 0.05 to 0.25 mg/mL. A volume of 10 mL from the diluted sample solution was mixed with 100 μL of the ANS solution and left to react for 15 min in the absence of light. Fluorescence measurements were taken using a spectrophotometer (LS-55, PerkinElmer, UK), with the ANS buffer serving as the baseline control. The instrument settings included an emission wavelength of 470 nm and an excitation wavelength of 390 nm. The relationship between the fluorescence intensity and the protein concentration was determined through linear regression analysis.

### 3.8. Emulsifying Properties

Initially, sunflower oil and PPI-polyphenol complexes were combined at a volume ratio of 1:3. A high-speed homogenizer (T25, IKA, Guangzhou scientific Instruments, Guangzhou, China 12,000 rpm, 1 min) was used to ensure thorough emulsification. Subsequently, 100 μL of the freshly prepared emulsion was introduced into 9.9 mL of sodium dodecyl sulfate solution (1 mg/mL). Lastly, the emulsion samples (0 min and 10 min) were measured at a wavelength of 500 nm. The following equations can calculate the emulsification activity index (EAI) and emulsion stability index (ESI):

$$ESI\left(min\right)=\frac{A_{10}}{A_{0}}\times 100\%$$

$$EAI\left(m^{2}/g\right)=\frac{2.303\times 2\times A_{0}\times N}{10000\times \varphi \times C\times L}$$

N, φ, L, and C are the dilution multiple (100), volume fraction of soybean oil (0.25), path length of the cuvette (1 cm), and concentration of PPI-polyphenol complexes (g/mL), respectively.

### 3.9. Interfacial Tension

The dynamic interfacial tension was measured using the pendant drop technique. This was facilitated by employing a drop shape analysis system (OT100, Ningbo NB Scientific Instruments, Ningbo, China) over a period of 1800 s.

### 3.10. Preparation of Emulsions

To prepare the emulsion, a blend was created by combining PPI-polyphenol complexes with sunflower oil at a volume ratio of 1:9. The total volume of emulsion was 20 mL. This mixture was promptly treated with a high-speed homogenizer (T25, IKA, Königswinter, Germany), running at 12,000 rpm for 3 min to obtain an emulsion.

### 3.11. Interfacial Protein Content (AP%) of Emulsion

The assessment of the AP of emulsions was conducted according to the research of Xu et al. [38]. Emulsions were centrifuged at a speed of 10,000 rpm for 30 min at 4 °C. The aqueous phase settled at the bottom was carefully aspirated using syringes. This aqueous phase was further refined by filtration through a membrane with a pore size of 0.44 µm. The AP was calculated according to the following equation:

$$AP\left(\%\right)=\frac{C_{0}-C_{i}}{C_{0}}\times 100$$

where C_0_ is the initial protein concentration of the emulsion, and C_i_ is the protein concentration of the filtrate.

### 3.12. Droplet Size and Zeta Potential

A laser diffraction particle size analyzer (Mastersizer 3000E, Malvern Panalytical, Malvern, UK) was employed to determine the mean droplet size. Meanwhile, the Zeta potential, which indicates the electrical charge of the droplets’ surface, was analyzed using a Zeta potential analyzer (NANOTRAC WAVE II, Microtrac MRB, Oklahoma City, OK, USA).

### 3.13. Confocal Laser Scanning Microscopy (CLSM)

CLSM (Zeiss 780, Leica Corp., Waldkirch, Germany) was utilized to visualize the microstructural characteristics of the emulsion. Nile red and Nile blue were applied as stains. Emulsions were observed using a laser at the excitation values of 488 and 633 nm.

### 3.14. Rheological Properties

The rheological characteristics of the emulsions were investigated utilizing a dynamic shear rheometer (DV3THA, Brookfield Corp., Middleboro, MA, USA). The frequency sweep was conducted over a range from 0.1 to 10 Hz at 25 °C. The storage modulus (G′) and the loss modulus (G″) were recorded.

### 3.15. Stability of Emulsion Measurements

#### 3.15.1. Emulsion Stability

The assessment of multiple light scattering for emulsions featuring distinct oil phases was conducted using a Turbiscan Tower instrument (Formulaction, Toulouse, France). Over a continuous period of 3 h, the device monitored and recorded changes in backscattering (ΔBS) and calculated the Turbiscan Stability Index (TSI).

#### 3.15.2. Oxidative Stability

The primary (PV) and secondary oxidation (TBARS) were quantified to evaluate the oxidation degree of the emulsion. These measurements were taken periodically throughout a 10-day accelerated storage trial, which was carried out at an elevated temperature of 55 °C.

### 3.16. Statistical Analysis

All experimental tests were conducted in triplicate. Statistical analysis of the results was performed utilizing the SPSS 20.0 software package. We applied Duncan’s test, setting the significance level at *p* < 0.05.

## 4. Conclusions

In this research, we explored the correlation between the structure of polyphenols and the stability of emulsions prepared by PPI-polyphenol complexes. As the hydroxyl group and carbon chain increased, the intensity of hydrogen bonding and hydrophobic interactions between PPI and the respective polyphenol was augmented. The binding of polyphenols induced a conformational expansion in PPI, concurrently reducing the interfacial tension. This facilitated the enhanced adsorption of PPI-polyphenol complexes at the oil–water interface, leading to the formation of a denser interfacial film, which effectively prevented the coalescence of oil droplets. The PPI-RA complex established a greater number of hydrogen bonds. This contributed to the generation of smaller droplet sizes and a more homogeneous emulsion structure. Furthermore, the PPI-RA-stabilized emulsion displayed superior rheological characteristics and oxidative stability. In conclusion, our research findings offered a novel perspective on selecting polyphenol types from a structural viewpoint to enhance PPI emulsification properties, offering a promising avenue for product innovation and development.

## Figures and Tables

**Table 1:** The compound structure, Ksv, Ka, n, and thermodynamic parameters (ΔH, ΔS, and ΔG) of PPI-RA and PPI-CF. RA is rosmarinic acid, CF is caffeic acid.

Sample	Structure	T (K)	Ksv (10^6^ M^−1^)	Ka (10^6^ M^−1^)	n	ΔH/ (kJ⋅mol^−1^)	ΔS/ (kJ⋅mol^−1^)	ΔG/ (kJ⋅mol^−1^)
**PPI-RA**	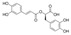	298	0.046	1.58	1.11	69.73	0.35	−35.17
		304	0.041	2.15	1.18	/	/	−37.27
		310	0.033	3.64	1.25	/	/	−39.39
**PPI-CF**	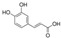	298	0.035	0.92	1.02	53.32	0.29	−33.91
		304	0.026	1.39	1.12	/	/	−35.45
		310	0.022	2.74	1.22	/	/	−37.20

**Table 2:** Emulsifying properties and AP% of PPI-RA and PPI-CF.

Sample	EAI (m^2^/g)	ESI (min)	AP (%)
PPI	83.95 ± 1.67 ^e^	68.58 ± 1.88 ^d^	39.52 ± 1.02 ^e^
PPI-30RA	95.47 ± 1.67 ^d^	89.59 ± 1.29 ^b^	49.67 ± 1.32 ^c^
PPI-50RA	105.54 ± 2.58 ^a^	94.68 ± 1.34 ^a^	56.31 ± 1.09 ^a^
PPI-70RA	101.68 ± 1.14 ^b^	90.35 ± 1.64 ^b^	52.22 ± 1.62 ^b^
PPI-30CF	92.64 ± 1.57 ^d^	82.11 ± 1.34 ^c^	42.69 ± 1.49 ^d^
PPI-50CF	98.67 ± 1.31^c^	90.73 ± 2.31 ^b^	54.28 ± 1.01 ^b^
PPI-70CF	95.64 ± 2.56 ^d^	85.64 ± 2.67 ^c^	48.36 ± 1.64 ^c^

Note: Comparisons were carried out between values in the same column, and values with a different letter(s) indicate a significant difference at *p* ≤ 0.05.

## Data Availability

The original contributions presented in this study are included in the article.
